# Serum lactate dehydrogenase level predicts the prognosis in bladder cancer patients

**DOI:** 10.1186/s12894-023-01239-0

**Published:** 2023-04-25

**Authors:** Shuo Gu, Chao Yang

**Affiliations:** Department of Urology, The Affiliated Huaian No.1 People’s Hospital of Nanjing Medical University, Jiangsu, 223300 China

**Keywords:** LDH, Bladder cancer, Survival, Progression, Prognostic marker

## Abstract

**Background:**

Recently, several studies investigated the association between lactate dehydrogenase (LDH) level and the prognosis of urothelial carcinoma. However, no studies explored the role of serum LDH level in the survival of overall bladder cancer (BC). In this study, we intended to address the association of LDH level with the prognosis of BC.

**Methods:**

206 patients with BC were included in this study. The clinical data and blood samples of patients were collected. The overall survival and progression-free survival were used. Kaplan–Meier method and Log rank test were used to evaluate the effects of LDH level on the survival of BC. Univariate and multivariate Cox regression analyses were utilized to identify prognosis predictors of BC.

**Results:**

Data indicated that serum LDH level in the BC patients was significantly higher than those in controls. In addition, this study suggested that serum LDH level was associated with T stage, N stage, tumor size, M stage, pathological type, and lymphovascular invasion. The Kaplan–Meier analysis found significant differences in the OS and PFS rate between lower and higher serum LDH level groups (LDH ≥ 225 U/L and < 225 U/L). Multivariate Cox regression indicated that pathological type, T2–3, and higher level of LDH were independently associated with adverse prognosis in BC patients.

**Conclusion:**

The higher serum LDH level (≥ 225 U/L) is associated with poor prognosis in patients with BC. Serum LDH level could be used as a novel predictive biomarker for BC patients.

**Supplementary Information:**

The online version contains supplementary material available at 10.1186/s12894-023-01239-0.

## Introduction

Bladder cancer (BC) is a prevalent malignancy and is the fourth most main malignancy in men [1]. More than 430,000 BC patients were diagnosed globally every year, which brings a heavy burden for the society [2]. A host of risk factors including cigarette smoking, alcohol intake, diets low in fruits and vegetables, urban living, occupational exposure to carcinogens, and genetic factors were associated with the development of BC [3]. Among these factors, cigarette smoking is the most primary risk factor for BC. It is estimated that tobacco was responsible for approximately 50% of all BC patients [4]. The most common symptom of BC is haematuria [5]. However, some BC patients with microscopic haematuria were not easily detected [6]; and there were no effective screening methods for BC, except for the cystoscope. Thus, detecting novel prognostic and diagnostic biomarkers is urgently required for patients with BC.

Most cancer cells utilize glucose metabolism by glycolysis to produce enough ATP to promote the development of cancer, which is served as the Warburg effect [7]. Among the enzymes participating in glycolysis, lactate dehydrogenase (LDH), an oxidoreductase, could converts pyruvate to lactate at the end of glycolysis; thereby, LDH is regarded as a promising molecular target for the development of new glycolytic inhibitors used in cancer therapy [8]. LDH may be a biomarker of tumor development due to its association with tumor metabolic changes [9].Urothelial carcinoma (UC) mainly consists of urothelial carcinoma of the bladder (UCB) and upper tract urothelial carcinoma (UTUC). Recently, a host of studies investigated the link between LDH level and the prognosis of UC [10–24]. Among these studies, no studies investigated the role of serum LDH level in the survival of overall BC; and previous studies mainly focused on UCB patients (the main type of BC). In this study, we intended to address the association of LDH level with clinicopathological markers of BC. In addition, we aimed to evaluate the ability of pretreatment serum LDH level in predicting the prognosis of BC patients.

## Methods

### Patients

This study was conducted after approval from the Ethics Committee of Huaian No.1 People’s Hospital. 206 consecutive BC patients were recruited between September 2014 and May 2017 from our Hospital. BC patients were divided into two groups, which was according to the pretherapeutic median levels of LDH. The diagnosis of BC patients was according to histological results. All relevant clinical and pathologic information was collected for each patient. Inclusion criteria for BC patients were as follows: one, patients with definite histologic evidence; two, patients older than 18 years; three, patients with complete data. Patients with other cancers, hepatitis, heart disease, and hemopathy were excluded. The informed consent form was provided by all subjects. This study was approved by the Ethics Committee of this hospital. This study was in consistent with the Declaration of Helsinki.

### Data collection and follow-up

Clinical information was collected from the medical records, including age, sex (male and female), smoking status (yes and no), T stage, N stage (N0 and N1-N3), tumor size (< 3 cm and > 3 cm), M stage (M0 and M1), lymphovascular invasion (LVI) status, perineural invasion (PNI) status, pathological type, and multifocality (Unifocal and Multifocal). Serum LDH levels were collected from all BC patients before treatment. The cut-off value of LDH level was defined as 225 U/L, which was the upper limit of normal range in this hospital. Thus, we defined the high LDH level group was over 225 U/L, and the low LDH level group was lower than 225 U/L. Overall survival (OS) was defined as the survival duration from the date of initial diagnosis to death due to any reasons or last follow-up. Progression-free survival (PFS) referred to the time for BC patients to survive without progression or death after treatment. The follow-up strategies were as follows: every two months for the first 2 years, quarterly for the next 2 years, and then semiannually for the remaining time thereafter. The follow-up time ended in June 2021.

### Statistical analysis

The correlation between serum LDH level and clinicopathological indexes was analyzed. We used the receiver operating characteristic (ROC) curve to assess the diagnostic ability of serum LDH level for detecting BC. The log-rank test was used to compare the OS and PFS between different groups. Univariate and Multivariate Cox regression analyses were conducted to identify prognostic variables associated with the survival of BC. Risk factors with a *P* value < 0.05 in univariate analysis were choose in further multivariate Cox regression analysis. 95% confidence interval (CI) and Hazard risk (HR) were figured out. Statistically significant Differences were set at *P* < 0.05. SPSS (version 19.0, Chicago, IL, USA), and Graphpad Prism (version 7.0, La Jolla, CA, USA), and MedCalc were used for statistical analyses.

## Results

### Clinicopathological variables of BC patients and LDH level

**Fig. 1:**
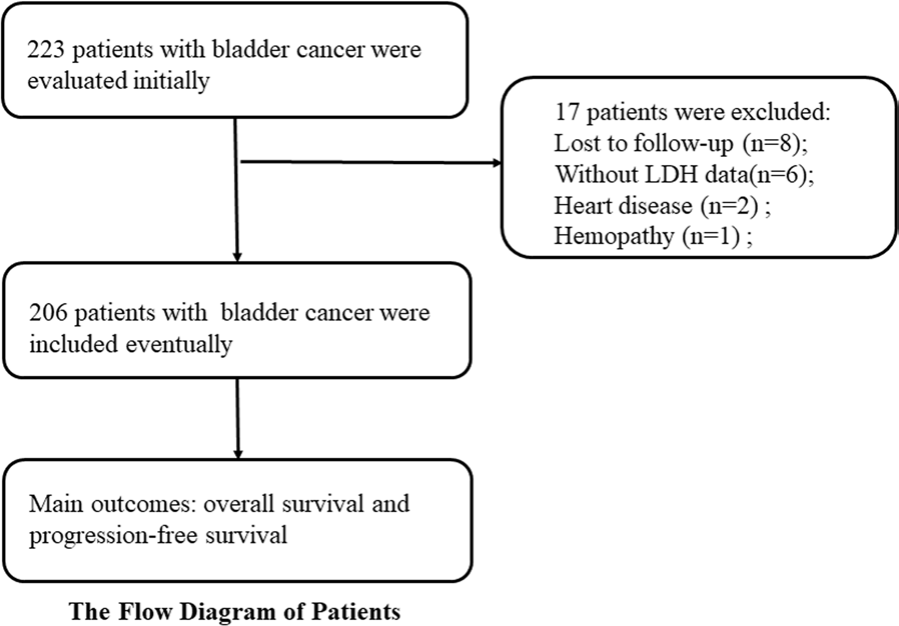
The flow diagram of patients

Clinicopathological variables for BC patients with LDH ≥ 225 U/L and < 225 U/L are shown in Table 1. 223 patients with BC were evaluated initially, and 206 cases were included finally (Fig. 1). Among the 206 BC patients, 149 cases were in the low LDH group and 57 cases were in the high LDH group. We found that LDH level was associated with T stage, N stage, tumor size, M stage, pathological type, and LVI status (Table 1). No significant differences were observed in two groups regarding age, sex, smoking status, PNI status, and multifocality status (Table 1).

**Table 1:** Demographics of 206 patients with the bladder cancer

Variables	Total patients (n = 206)	Groups		*P*
		Low LDH	High LDH	
		< 225 U/L	≥ 225 U/L	
Median age (years)			57	0.073
< 64	93 (45.1%)	73 (49.0%)	20 (35.1%)	
≥ 64	113 (54.9%)	76 (51.0%)	37 (64.9%)	
Sex, n%				0.709
Male	159 (77.2%)	114 (76.5%)	45 (78.9%)	
Female	47 (22.8%)	35 (23.5%)	12 (21.1%)	
Smoking				0.592
Yes	95 (46.1%)	67 (45.0%)	28 (49.1%)	
No	111 (53.9%)	82 (55.0%)	29 (50.9%)	
Pathological type				**< 0.001**
Transitional cell carcinoma	188 (91.3%)	144 (96.6%)	44 (77.2%)	
Squamous cell carcinoma	12 (5.8%)	4 (2.7%)	8 (14.0%)	
Adenocarcinoma	6 (2.9%)	1 (0.7%)	5 (8.8%)	
T stage				**< 0.001**
Ta, Tis, T1	122 (59.2%)	101 (67.8%)	21 (36.8%)	
T2–3	84 (40.8%)	48 (32.2%)	36 (63.2%)	
N stage				**< 0.001**
N0	183 (88.8%)	144 (96.6%)	39 (68.4%)	
N1–3	23 (11.2%)	5 (3.4%)	18 (31.6%)	
M stage				**< 0.001**
M0	188 (91.3%)	146 (98.0%)	42 (73.7%)	
M1	18 (8.7%)	3 (2.0%)	15 (26.3%)	
Tumor size (cm)				**0.011**
< 3 cm	55 (26.7%)	47 (31.5%)	8 (14.0%)	
> 3 cm	151 (73.3%)	102 (68.5%)	49 (86.0%)	
LVI				**< 0.001**
Present	76 (36.9%)	43 (28.9%)	33 (57.9%)	
Absent	130 (63.1%)	106 (71.1%)	24 (42.1%)	
PNI				0.075
Present	29 (14.1%)	17 (11.4%)	12 (21.1%)	
Absent	177 (85.9%)	132 (88.6%)	45 (78.9%)	
Multifocality				0.559
Unifocal	161 (78.2%)	118 (79.2%)	43 (75.4%)	
Multifocal	45 (21.8%)	31 (20.8%)	14 (24.6%)	

*LVI* lymphovascular invasion, *PNI* perineural invasion, *LDH* lactate dehydrogenase

Bold values are statistically significant (*P* < 0.05)

### Diagnostic value of LDH level for detecting BC

**Fig. 2:**
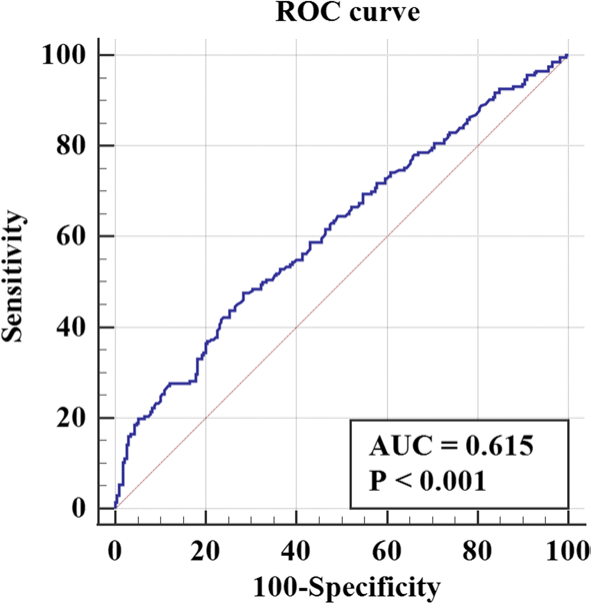
The receiver operating characteristic curve of LDH level for detecting bladder cancer

230 controls were included in this study. The detailed information for controls is summarized in Additional file 2: Table S1. We measured the serum level of LDH in BC patients and controls, and found that the LDH level in BC group were significantly higher than those in control group. We used the ROC curve to evaluate the diagnostic function of serum LDH level for BC (Fig. 2). The sensitivity and specificity were 47.57% and 71.74% with a cutoff value of 211.53 (Additional file 3: Table S2), respectively. The area under the curve was 0.615 (95%CI: 0.567–0.661, *P* < 0.001), suggesting that the diagnostic ability of serum LDH level for detecting BC was relatively moderate.

### Correlation between LDH level and the prognosis in patients with BC

**Fig. 3:**
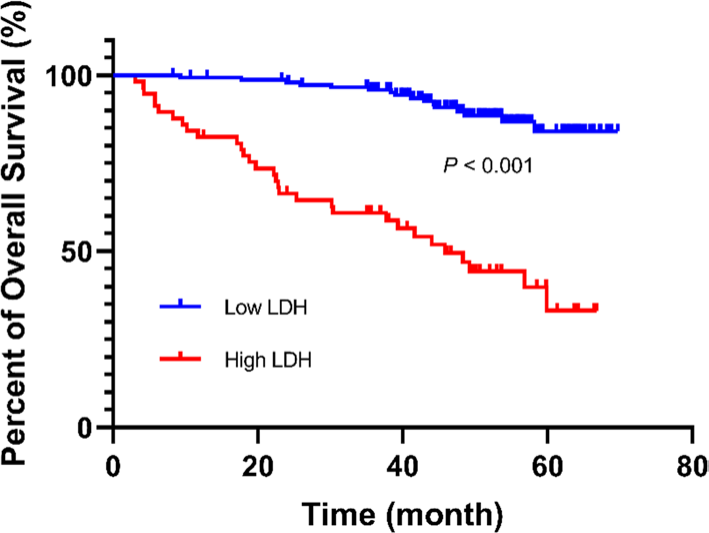
Comparison of overall survival rate between high LDH level group and low LDH level group

**Fig. 4:**
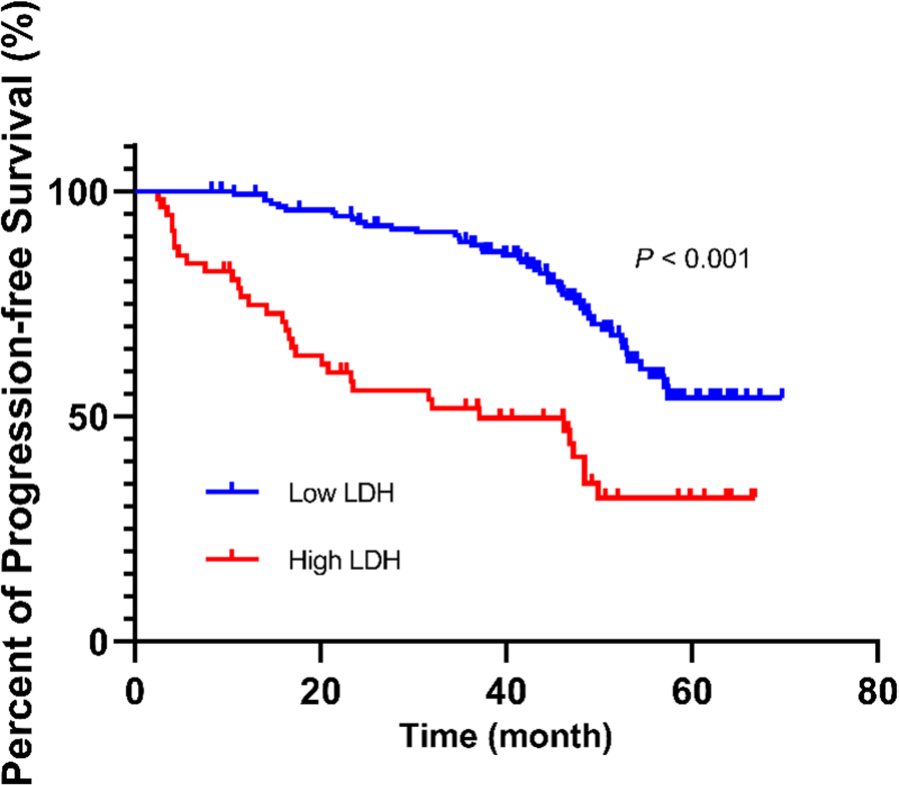
Comparison of progress-free survival rate between high LDH level group and low LDH level group

We divided all BC patients into two groups (LDH ≥ 225 U/L and < 225 U/L) according to the level of LDH. In order to evaluate the prognostic value of LDH in BC, Kaplan–Meier analysis was utilized to assess the association between LDH level and follow-up data, and the log-rank test was mainly used for analyzing statistical difference. The results showed that the high level of LDH was negatively correlated with OS and PFS (Figs. 3, 4), suggesting that the survival rate of BC patients with lower level of LDH were significantly higher than those with higher level of LDH. In addition, we compared the survival rates between UCB group and non-UCB group (squamous cell carcinoma and adenocarcinoma). Data indicated that the survival rate of BC patients with UCB was significantly higher than that in non-UCB group (Additional file 1: Figure S1 and S2).

### Identification of prognostic factors of OS and PFS

Univariate and multivariate Cox analyses were used to identify prognostic factors of BC patients. Univariate analysis showed that older age, T2–3 stage, N1–3 stage, M1 stage, LVI, PNI, non-UCB, and higher level of LDH were significantly associated with reduced OS (Table 2), while smoking, T2–3 stage, N1–3 stage, M1 stage, tumor size ≥ 3 cm, LVI, PNI, non-UCB, and higher level of LDH negatively correlated with PFS (Table 3). Multivariate analysis indicated that T2–3 stage, non-UCB, and higher level of LDH were independent predictive factors for worse OS and PFS. In addition, we identified prognostic factors for BC patients with UCB, and found that T2–3 stage, and higher LDH level were prognostic factors for worse OS (Additional file 4: Table S3). Furthermore, we observed that T2–3 stage, M1 stage, tumor size ≥ 3 cm were adverse factors for PFS in UCB (Additional file 5: Table S4).

**Table 2:** Univariate and multivariate cox regression analysis for overall patient survival

Factors	Overall survival			
	Univariate		Multivariate	
	HR (95%CI)	*P* value	HR (95%CI)	*P* value
Age				
≥ 64 vs. < 64 years	**3.48 (1.69–7.21)**	**0.001**	1.28 (0.57–2.87)	0.547
Sex				
Male vs. female	0.88 (0.46–1.70)	0.713	–	–
Smoking				
Yes vs. no	1.43 (0.80–2.54)	0.226	–	–
Pathological type				
Squamous cell carcinoma/adenocarcinoma vs. transitional cell carcinoma	**16.91 (8.69–32.90)**	**< 0.001**	**4.50 (1.96–10.30)**	**< 0.001**
T stage				
T2–3 vs. Ta, Tis, T1	**8.02 (3.75–17.18)**	**< 0.001**	**3.37 (1.41–8.10)**	**0.007**
N stage				
N1–3 vs. N0	**13.92 (7.65–25.32)**	**< 0.001**	1.74 (0.45–6.72)	0.420
M stage				
M1 vs. M0	**14.77 (7.94–27.46)**	**< 0.001**	1.25 (0.39–3.99)	0.704
Tumor size				
≥ 3 vs. < 3 cm	1.64 (0.79–3.40)	0.182	–	–
LVI				
Present vs. absent	**4.74 (2.54–8.87)**	**< 0.001**	1.53 (0.66–3.55)	0.327
PNI				
Present vs. absent	**3.04 (1.60–5.76)**	**0.001**	1.13 (0.46–2.78)	0.783
Multifocality				
Multifocal vs. unifocal	1.04 (0.53–2.05)	0.900	–	–
LDH				
High vs. low	**7.23 (3.94–13.24)**	**< 0.001**	**3.13 (1.55–6.33)**	**0.002**

*LVI* lymphovascular invasion, *PNI* perineural invasion, *LDH* lactate dehydrogenase

Bold values are statistically significant (*P* < 0.05)

**Table 3:** Univariate and multivariate cox regression analysis for progression-free survival

Factors	Progression-free survival			
	Univariate		Multivariate	
	HR (95%CI)	*P* value	HR (95%CI)	*P* value
Age				
≥ 64 vs. < 64 years	1.03 (0.66–1.60)	0.910	–	–
Sex				
Male vs. female	1.52 (0.84–2.76)	0.165	–	–
Smoking				
Yes vs. no	**1.59 (1.02–2.48)**	**0.041**	1.46 (0.92–2.32)	0.110
Pathological type				
Squamous cell carcinoma/adenocarcinoma vs. transitional cell carcinoma	**9.12 (4.76–17.49)**	**< 0.001**	**3.07 (1.37–6.88)**	**0.007**
T stage				
T2–3 vs. Ta, Tis, T1	**2.69 (1.72–4.22)**	**< 0.001**	1.66 (0.98–2.81)	0.061
N stage				
N1–3 vs. N0	**13.67 (7.58–24.65)**	**< 0.001**	3.12 (0.84–11.60)	0.090
M stage				
M1 vs. M0	**18.37 (9.42–35.84)**	**< 0.001**	3.02 (0.89–10.28)	0.077
Tumor size				
≥ 3 vs. < 3 cm	**3.20 (1.60–6.42)**	**0.001**	**2.81 (1.37–5.78)**	**0.005**
LVI				
Present vs. absent	**1.82 (1.17–2.83)**	**0.008**	0.86 (0.47–1.55)	0.604
PNI				
Present vs. absent	**2.18 (1.22–3.91)**	**0.008**	1.07 (0.49–2.35)	0.870
Multifocality				
Multifocal vs. unifocal	1.01 (0.60–1.71)	0.978	–	–
LDH				
High vs. low	**3.08 (1.96–4.83)**	**< 0.001**	**1.70 (1.00–2.87)**	**0.049**

*LVI* lymphovascular invasion, *PNI* perineural invasion, *LDH* Lactate dehydrogenase

Bold values are statistically significant (*P* < 0.05)

## Discussion

This study suggested that serum LDH level was associated with T stage, N stage, tumor size, M stage, pathological type, and LVI status. Serum LDH level had a moderate diagnostic value in detecting BC patients. Multivariate Cox regression indicated that non-UCB, T2–3, and higher level of LDH were independently associated with adverse prognosis in BC patients (including OS and PFS). Therefore, this study provided evidence that higher LDH level was associated with worse prognosis in BC and could be a prognostic indicator of BC patients.

Hannisdal et al. from Denmark first showed that UCB patients with LDH > 400 U/L showed shorter survival comparing to those with LDH < 400 U/L [11]. Two Japanese studies found that higher LDH level was negatively associated with the OS of UCB after radical cystectomy [12] or postcystectomy recurrent UCB [13]. However, a study by Yang et al. from Taiwan indicated that serum LDH level was not related with the disease-specific survival of UCB in their population [10], which was not consistent with previous findings [11–13]. We assumed the following factors may provide evidence for these conflicting points. One, the results of Taiwanese study was obtained by the univariate analysis [10], which may underpower the credibility of results. Two, clinical heterogeneity should not be ignored. Two Japanese studies investigated the recurrent UCB [12, 13], while the Taiwanese study explored invasive UCB [10]. Three, their indicators evaluating survival differed, such as OS or disease-free survival (DFS). Four, their varied treatment therapies may also contribute to their contradictory results concerning the survival. It is of note that Yang et al. conducted another study [14], and found that serum LDH level ranging from 200 to 300 U/L was an independent factor associated with UTUC after multivariate analysis, but not for UCB. Obviously, the tumor location may be a crucial reason for explaining their contradictory results by Yang et al. [10, 14] Last but not least, the cut-off values of serum LDH level were diverse, which may exert effects on the final results.

Regarding UTUC, other studies [15–17] also delineated the association between serum LDH level and the prognosis of UTUC. Zhang et al. showed that preoperative serum LDH level was as a negative predictor of OS and DFS [15], while Tan et al. indicated that preoperative LDH was not an independent prognostic indicator for patients with UTUC [15]. However, Tan et al. suggested that elevated LDH level correlated with worse OS in UTUC patients with localized disease [16]. Kluth et al. also indicated that serum LDH level was not associated with the survival of UTUC [18]. As for the Japanese study by Ito et al., they observed that LDH ≥ 210 IU/L were significantly related with extraurothelial recurrence in N_0_M_0_ patients with renal pelvic cancer [17], but not OS or DFS.

Besides UCB and UTUC, some researchers shed light on the investigation of serum LDH level and UC survival. Sengelov et al. showed that LDH level were related significantly to the survival of UC by univariate analyses [19], similar to the findings by Japanese studies via multivariate analyses [20–22]. In addition, two studies from Japan [23] and Spain [24] indicated that serum LDH level was not a prognostic factor in UC patients. We hypothesized that the negative results in the Spanish study [24] may due to its limited sample size (only 56 UC cases). Additionally, they utilized univariate analyses to obtain these results [24], which underpowered the reliability of their results.

Due to these conflicting findings concerning UCB, UTUC or UC, Wu et al. conducted a meta-analysis to address this issue [25]. They suggested that a high pretreatment serum LDH level was linked with an inferior OS, cancer-specific survival, and DFS in UC patients [25]. Subgroup analyses revealed that high serum LDH level was associated with a poor OS and DFS in UTUC, and a short OS in UCB [25]. It is noticeable that the meta-analysis by Wu et al. did not include a study [18], which was in line with inclusion criteria of this meta-analysis. Another meta-analysis by Zhang et al. also indicated that a high LDH level was associated with an adverse prognosis in many solid tumors [26].

In this study, we included 206 BC patients, and found that a higher pretreatment serum LDH level was associated with an unfavorable prognosis in BC patients. Abovementioned studies primarily investigated the survival of UCB patients (the main type of BC), while this study explored the prognosis of overall BC with UCB, squamous cell carcinoma, and adenocarcinoma. We compared the survival rates between UCB group and non-UCB group, and found that the survival rate of BC patients with UCB was significantly higher than that in non-UCB group, which was not investigated in other studies. Furthermore, multivariate analysis indicated that pathological type (non-UCB vs UCB) was an independent predictive factor for worse OS and PFS.

This study had several limitations. First, the sample size of this study was not large enough. Second, some confounding factors affecting the survival of BC may not be investigated in this study, thereby exerting effects on final results. Third, the findings observed by this study were only yielded in one single center; thus, multi-center studies are urgently needed to verify these findings. Fourth, UTUC patients were not investigated in this study. Fifth, diverse treatment strategies may affect the survival analysis, thereby interfering the effect of serum LDH level on the prognosis of BC patients. Last, regarding for the ROC curve (Fig. 2), the AUC value was 0.615, indicating that there was no clear cut differentiation. High LDH level might just be another marker for poor performance.

## Conclusions

Totally, this study observes that a higher pretreatment serum LDH level is associated with adverse prognosis in BC patients. In addition, serum LDH level may predict the occurrence of BC patients. Thus, LDH level could be served as a prognostic biomarker for BC patients.

## Supplementary Information

**Additional file 1. Supplementary Figure 1.** Comparison of overall survival rate between UCB group and non-UCB group. **Supplementary Figure 2.** Comparison of progress-free survival rate between UCB group and non-UCB group.

**Additional file 2. Supplement Table 1.** Demographics between BC patients and controls.

**Additional file 3. Supplementary Table 2.** ROC curves for predictive values of serum LDH level in UCB.

**Additional file 4. Supplementary Table 3.** Univariate and multivariate cox regression analysis for overall patient survival in transitional cell carcinoma.

**Additional file 5. Supplementary Table 4.** Univariate and multivariate cox regression analysis for progression-free survival in transitional cell carcinoma.

## Data Availability

All data generated or analyzed during this study are included in this published article [and its supplementary information files].
