# Supplementary material for: Serum lactate dehydrogenase level predicts the prognosis in bladder cancer patients
Source: BMC Urol. 2023 Apr 25;23:65. doi: 10.1186/s12894-023-01239-0 (PMC10127081; doi:10.1186/s12894-023-01239-0)
Supplement: Supplementary file 1 — Additional file 1. Supplementary Figure 1. Comparison of overall survival rate between UCB group and non-UCB group. Supplementary Figure 2. Comparison of progress-free survival rate between UCB group and non-UCB group. [file 12894_2023_1239_MOESM1_ESM.docx]

**Supplementary Figure** **legends**


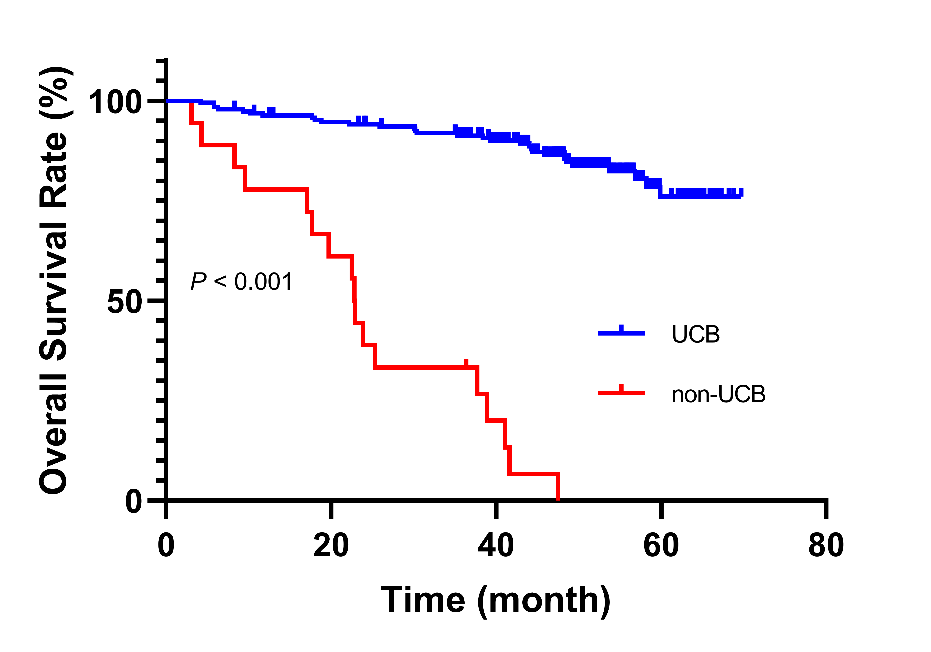


**Supplementary Figure 1.** Comparison of overall survival rate between UCB group and non-UCB group.


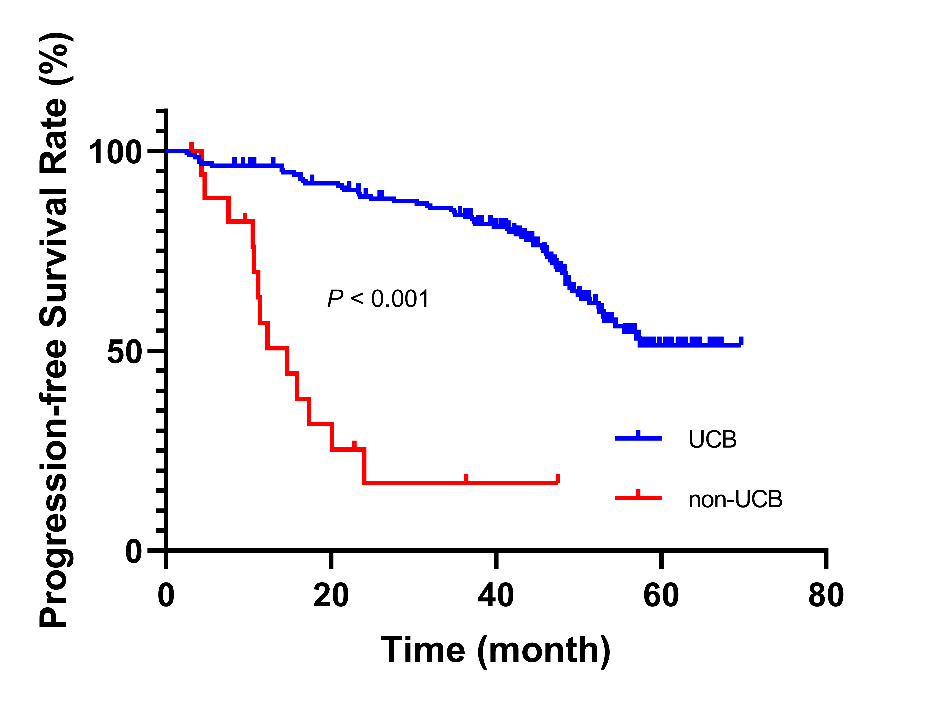


**Supplementary Figure 2.** Comparison of progress-free survival rate between UCB group and non-UCB group.
