# Supplementary material for: Serum lactate dehydrogenase level predicts the prognosis in bladder cancer patients
Source: BMC Urol. 2023 Apr 25;23:65. doi: 10.1186/s12894-023-01239-0 (PMC10127081; doi:10.1186/s12894-023-01239-0)
Supplement: Supplementary file 2 — Additional file 2. Supplement Table 1. Demographics between BC patients and controls. [file 12894_2023_1239_MOESM2_ESM.docx]

**Supplement Table 1.** Demographics between BC patients and controls.

| Variables | BC patients | Controls | *P* |
| --- | --- | --- | --- |
|  | (n=206) | (n=230) |  |
| Median age (years) |  |  | 0.311 |
| < 64 | 93 (45.1%) | 115 (50.0%) |  |
| ≥ 64 | 113 (54.9%) | 115 (50.0%) |  |
| Sex |  |  | 0.787 |
| Male | 159 (77.2%) | 175 (76.1%) |  |
| Female | 47 (22.8%) | 55 (23.9%) |  |
| Smoking |  |  | 0.711 |
| Yes | 95 (46.1%) | 102 (44.3%) |  |
| No | 111 (53.9%) | 128 (55.7%) |  |
| Serum LDH (U/L), IQR | 209.45 (193.88, 226.15) | 200.55 (185.73, 213.83) | **<0.001** |

BC, bladder cancer; IQR, interquartile range. Bold values are statistically significant (*P* < 0.05).
