# Supplementary material for: Serum lactate dehydrogenase level predicts the prognosis in bladder cancer patients
Source: BMC Urol. 2023 Apr 25;23:65. doi: 10.1186/s12894-023-01239-0 (PMC10127081; doi:10.1186/s12894-023-01239-0)
Supplement: Supplementary file 3 — Additional file 3. Supplementary Table 2. ROC curves for predictive values of serum LDH level in UCB. [file 12894_2023_1239_MOESM3_ESM.docx]

**Supplementary Table 2.** ROC curves for predictive values of serum LDH level in UCB.

| LDH (pg/ml) | Youden index J | Associated criterion | Sensitivity % | Specificity % | AUC (95%) | 95% CI | Significance level *P* (Area=0.5) |
| --- | --- | --- | --- | --- | --- | --- | --- |
| Serum LDH | 0.1931 | > 211.5 | 47.57 | 71.74 | 0.615 | 0.567 to 0.661 | <0.001 |

ROC, Receiver Operating Characteristic; UCB, Urothelial Carcinoma of the Urinary Bladder; AUC, Area Under the ROC Curve; 95% CI, Confidence interval.
