# Supplementary material for: Serum lactate dehydrogenase level predicts the prognosis in bladder cancer patients
Source: BMC Urol. 2023 Apr 25;23:65. doi: 10.1186/s12894-023-01239-0 (PMC10127081; doi:10.1186/s12894-023-01239-0)
Supplement: Supplementary file 4 — Additional file 4. Supplementary Table 3. Univariate and multivariate cox regression analysis for overall patient survival in transitional cell carcinoma. [file 12894_2023_1239_MOESM4_ESM.docx]

**Supplementary Table 3.** Univariate and multivariate cox regression analysis for overall patient survival in transitional cell carcinoma.

| Factors | Overall survival | | | | | | |
| --- | --- | --- | --- | --- | --- | --- | --- |
|  | Univariate | | Multivariate | | | | |
|  | HR (95%CI) | *P*-value | | | HR (95%CI) | | *P*-value |
| Age |  | | |  | |  |  |
| ≥ 64 vs. < 64 years | **4.28(1.64-11.19)** | | | **0.003** | | 2.54(0.91-7.05) | 0.074 |
| Sex |  | | |  | |  |  |
| Male vs. female | 0.99(0.42-2.30) | | | 0.976 | | - | - |
| Smoking |  | | |  | |  |  |
| Yes vs. no | 1.93(0.92-4.05) | | | 0.083 | | - | - |
| T stage |  | | |  | |  |  |
| T2-3 vs. Ta, Tis, T1 | **7.16(2.92-17.52)** | | | **<0.001** | | **3.05(1.11-8.34)** | **0.030** |
| N stage |  | | |  | |  |  |
| N1-3 vs. N0 | **15.88(7.45-33.84)** | | | **<0.001** | | 6.10(1.00-37.08) | 0.050 |
| M stage |  | | |  | |  |  |
| M1 vs. M0 | **16.89(7.55-37.77)** | | | **<0.001** | | 1.47(0.39-5.61) | 0.574 |
| Tumor size |  | | |  | |  |  |
| ≥ 3 vs. < 3cm | 0.93(0.43-2.03) | | | 0.854 | | - | - |
| LVI |  | | |  | |  |  |
| Present vs. absent | **4.24(1.98-9.07)** | | | **<0.001** | | 1.74(0.66-4.59) | 0.261 |
| PNI |  | | |  | |  |  |
| Present vs. absent | **3.57(1.63-7.80)** | | | **0.001** | | 0.36(0.10-1.32) | 0.123 |
| Multifocality |  | | |  | |  |  |
| Multifocal vs. unifocal | 1.24(0.55-2.78) | | | 0.604 | | - | - |
| LDH |  | | |  | |  |  |
| High vs. low | **6.04(2.91-12.55)** | | | **<0.001** | | **2.80(1.25-6.30)** | **0.013** |

LVI, lymphovascular invasion; PNI, perineural invasion, LDH, Lactate dehydrogenase.

Bold values are statistically significant (*P* < 0.05).
