# Supplementary material for: Serum lactate dehydrogenase level predicts the prognosis in bladder cancer patients
Source: BMC Urol. 2023 Apr 25;23:65. doi: 10.1186/s12894-023-01239-0 (PMC10127081; doi:10.1186/s12894-023-01239-0)
Supplement: Supplementary file 5 — Additional file 5. Supplementary Table 4. Univariate and multivariate cox regression analysis for progression-free survival in transitional cell carcinoma. [file 12894_2023_1239_MOESM5_ESM.docx]

**Supplementary Table 4.** Univariate and multivariate cox regression analysis for progression-free survival in transitional cell carcinoma.

| Factors | Progression-free survival | | | | |
| --- | --- | --- | --- | --- | --- |
|  | Univariate | | | Multivariate | |
|  | HR (95%CI) | *P*-value | | HR (95%CI) | *P*-value |
| Age |  |  |  | |  |
| ≥ 64 vs. < 64 years | 0.93(0.57-1.51) | 0.767 | - | | - |
| Sex |  |  |  | |  |
| Male vs. female | 1.70(0.87-3.34) | 0.122 | - | | - |
| Smoking |  |  |  | |  |
| Yes vs. no | **1.97(1.20-3.23)** | **0.007** | 1.51(0.91-2.50) | | 0.108 |
| T stage |  |  |  | |  |
| T2-3 vs. Ta, Tis, T1 | **2.32(1.43-3.77)** | **0.001** | **1.75(1.01-3.03)** | | **0.048** |
| N stage |  |  |  | |  |
| N1-3 vs. N0 | **13.85(6.92-27.70)** | **<0.001** | 2.05(0.58-12.22) | | 0.205 |
| M stage |  |  |  | |  |
| M1 vs. M0 | **21.73(9.68-48.79)** | **<0.001** | **5.98(1.47-24.30)** | | **0.012** |
| Tumor size |  |  |  | |  |
| ≥ 3 vs. < 3cm | **2.68(1.33-5.41)** | **0.006** | **3.04(1.45-6.37)** | | **0.003** |
| LVI |  |  |  | |  |
| Present vs. absent | 1.46(0.89-2.39) | 0.136 | - | | - |
| PNI |  |  |  | |  |
| Present vs. absent | **2.38(1.27-4.48)** | **0.007** | 1.08(0.45-2.60) | | 0.868 |
| Multifocality |  |  |  | |  |
| Multifocal vs. unifocal | 1.10(0.63-1.93) | 0.739 | - | | - |
| LDH |  |  |  | |  |
| High vs. low | **2.45(1.47-4.07)** | **0.001** | 1.42(0.79-2.54) | | 0.240 |

LVI, lymphovascular invasion; PNI, perineural invasion, LDH, Lactate dehydrogenase.

Bold values are statistically significant (*P* < 0.05).
